# Can artificial intelligence bridge critical gaps in hypertension prediction and risk assessment in low- and middle-income countries? A scoping review

**DOI:** 10.1136/bmjph-2025-003435

**Published:** 2026-05-22

**Authors:** David Sasu, Tsatsu Agbettor, Angela Owusu Ansah, William Annoh, Lecia Ackerson, Ayeyi Adjoa Domeyo Ohene-Adu, Roselyn Sackitey-Matey

**Affiliations:** 1 Ashesi University, Berekuso, Ghana; 2 Academic Affair, Ashesi University, Berekuso, Ghana

**Keywords:** Public Health, Risk Assessment, Digital Technology

## Abstract

**Objective:**

According to the WHO, as of 2025, 1.4 billion adults between the ages 30–79 were living with hypertension, with approximately two-thirds of that number from low- and middle-income countries (LMICs). By 2025, the number of individuals with hypertension is expected to have reached 1.5 billion, with sub-Saharan Africa alone projected to have 74.7 million cases. In recent years, artificial intelligence (AI) has demonstrated the potential to improve the accuracy of risk assessment tools for predicting, managing and diagnosing diseases before they escalate. This paper aims to conduct a scoping review to identify current research, innovations and developments in the application of AI-based tools for hypertension prediction and risk assessment, specifically in LMICs.

**Design:**

This scoping review used the Preferred Reporting Items for Systematic Reviews and Meta-Analyses extension for Scoping Reviews guidelines to ensure proper representation and organisation of the literature. A search was conducted across multiple databases, including PubMed, Elsevier, Google Scholar, Scopus, IEEE Xplore and the ACM Digital Library. Two independent reviewers used the search terms in Appendix A to identify relevant literature. Initial scans were followed by a detailed evaluation based on well-structured and clear inclusion and exclusion criteria. A third, more experienced reviewer resolved any selection discrepancies flagged from the first two reviews.

**Data sources:**

The literature that would be included within this study will primarily be sourced from the following scientific literature repositories: PubMed, Scopus, Elsevier, Google Scholar, IEEE and ACM Digital Library.

**Eligibility criteria:**

The inclusion and exclusion criteria defined and used to guide the literature selection process within this review included the following:

1. The literature should primarily be about the study of the deployment of AI algorithms or tools in the diagnosis, detection or risk assessment of hypertension in an LMIC.
2. The data used in the literature to train and assess any implemented AI algorithms should be obtained from either an LMIC.
3. The literature should have been published within the last 10 years, that is, 2014–2025.

**Data extraction and synthesis:**

The scoping review process followed the steps first outlined by Arksey and O’Malley in 2005. These steps are:

1. Identifying the research question.
2. Identifying the relevant studies within the literature.
3. Selecting the studies within the relevant studies that most align with the research question.
4. Charting the data that is present within these selected studies to get a deeper understanding of them.
5. Collating, summarising and reporting the information found in the selected literature.

From an initial result of 1371 papers, 5 were selected based on predefined inclusion and exclusion criteria. These studies solely focused on AI-based risk assessment and prediction of hypertension in LMICs.

**Results:**

The review identified significant research on AI applications for hypertension risk assessment and prediction in LMICs. Notable studies include an Ethiopian study on using machine learning algorithms, achieving an accuracy of 88.81%, precision of 89.62% and recall of 97.04% in hypertension risk prediction. Another research study involving hypertensive patients from South Asian countries (Bangladesh, Nepal, India) employed non-invasive information and machine learning tools for accurate hypertension prediction. The reported prediction accuracy of the studies found ranged from 78% to 97% depending on dataset size and AI model used. Algorithms discussed in these papers include logistic regression, decision trees and naïve Bayes.

**Conclusions:**

The reviewed studies highlight the promising potential of AI to enhance hypertension prevention and management in LMICs. Successful implementation of AI tools requires the development of locally contextualised models and the availability of validated local data. AI can significantly impact hypertension outcomes in LMICs, provided these conditions are met.

**WHAT IS ALREADY KNOWN ON THIS TOPIC**

- Hypertension is a global health concern, with two-thirds of all hypertensive individuals coming from low- and middle-income countries (LMICs). To reduce the high incidence of hypertension within LMICs, several digital solutions have been created; however, there is a lack of implementation due to poor digital infrastructure, relatively high costs for these patients as well as little to no digital infrastructure.

**WHAT THIS STUDY ADDS**

- This study conducts a scoping review on the existing application of artificial intelligence (AI) in hypertension prediction and risk assessment in LMICs, identifying successful implementations, for instance, machine learning models used in Kenya and Bangladesh, which showed a high accuracy of hypertension prediction and risk assessment. It also identifies major challenges obstructing AI adoption in LMICs, as well as gives solutions that would make integration more feasible.

**HOW THIS STUDY MIGHT AFFECT RESEARCH, PRACTICE OR POLICY**

- Policymakers can use these insights to prioritise investments in AI tools in the healthcare sector, digital health infrastructure, create policies for ethical AI implementation and develop training programmes for healthcare professionals in LMICs.

## Introduction

According to the WHO, hypertension occurs when an individual’s blood pressure (BP) is 140/90 mm Hg or higher.^1^ As of 2025, 1.4 billion adults between 30 and 79 years old are living with hypertension, with two-thirds of these individuals mainly from low- and middle-income countries (LMICs).^2^ Unfortunately, hypertension has become a key factor in premature deaths worldwide.^2^ The impact of hypertension has not only affected the healthcare systems of LMICs but has also resulted in significant economic challenges for these countries. The cost of treating cardiovascular diseases (CVDs) such as hypertension in LMICs between 2011 and 2015 was over US$3.7 trillion, which accounts for almost 2% of LMICs’ gross domestic product.^3^ Diseases such as this have resulted in an estimated cumulative economic loss of US$7.28 trillion and an annual loss of US$500 billion by LMICs from 2011 to 2015.^3^

In an effort to reduce the high incidence of hypertension within these regions, several approaches have been used, including different cost-effective technological tools that have been implemented over the years. Leveraging the ubiquitous nature of mobile phone technology within LMICs to combat hypertension, various health applications such as mobile telemedical software have been deployed in the past with some success.^4^ However, such technological solutions’ usability has often been questioned since their implementation is usually fraught with peculiar challenges. For instance, these technological solutions are sometimes viewed as unsustainable, due to their reliance on the use of hardware devices, which may be expensive to acquire or maintain.^5^ The process of setting up a mHealth (Mobile) system in rural Tanzania, for example, was met with significant challenges, with locals being unable to afford the cost of airtime needed for SMS text messaging.^6^ A study in Honduras and Mexico attempted to increase patients’ use of a home BP monitor with automated self-management calls using a cloud computing model. Unfortunately, one of the limitations of the study was that these monitors are not readily accessible in LMICs, with patients unable to afford battery replacements for these devices.^4^

Unfortunately, hypertension may only be recognised when BP is checked, hence the strong likelihood of those with it being unaware of the symptoms. In fact, 44% of adults do not know they have hypertension.^1^ There have been efforts to improve early-stage hypertension detection with risk assessment tools. In Shanghai, researchers developed a simple, valid and accurate screening tool to identify persons with increased risks of hypertension based on risk factors such as age, family history of high BP, BMI, abdominal obesity, etc.^7^ In the Americas, a calculator for CVD risk and hypertension management, ie, the HEARTS app, was built. The HEARTS app helps individuals and health professionals improve their understanding of CVD risk detection, providing them with more information on the necessary risk factors as well as simplified interventions to manage hypertension.^8^

Even though traditional risk assessment tools based on statistical methods have been demonstrated to show good performance in adequately assessing hypertension risk, risk assessment tools powered by artificial intelligence (AI) algorithms have been recorded in recent times to have greater levels of accuracy. AI is a technology that gives computers and machines the ability to mimic human intelligence and solve complex problems.^9^

A comparative analysis paper by Yazdi *et al* reveals that AI can significantly enhance the disease risk assessment processes, offering rapid and detailed insights. This paper concludes that integrating AI into the disease risk assessment process could be a game-changer.^10^

Using AI presents the opportunity to produce an early diagnosis of hypertension. In more developed countries, AI is being used efficiently to predict, manage and diagnose diseases before they escalate.^10^ One unique feature of AI is its ability to make decisions and take actions that will lead to the best possible output.^11^ For example, using a deep neural network, Barker *et al* developed an AI-Enabled ECG (AI-ECG) that delivers tailored hypertensive (HPT) risk assessments, early intervention and remote disease monitoring in hypertension care for patients.^12^ Soh *et al* developed a machine learning-based intelligence tool for hypertension detection using Empirical Mode Decomposition. The tool automates the classification of HPT versus normal ECG signals, aiding in early detection and management of hypertension.^13^

While AI has been globally discussed in research and academia, only a few scoping reviews exist within the current literature, identifying current work, research and innovations regarding the development and application of AI-based tools in the prediction and risk assessment of hypertension in LMICs. The prevalence of hypertension in LMICs is a rising concern, making this review imperative as it would help identify the gaps within this research area, as well as inform the creation of AI-based hypertension diagnostic tools.

## Methods

The research study uses the Preferred Reporting Items for Systematic Reviews and Meta-Analyses Extension for Scoping Reviews statement on reporting items for scoping reviews.^14^ This research framework helps in the proper representation and organisation of the broad literature that is available for the research topic. The following search databases were used for this review: PubMed, Elsevier, Google Scholar, Scopus, IEEE and the ACM Digital Library. After these databases were searched, only articles that were published from January 2014 to August 2025 and written in English were included in the list of articles considered for this scoping review.

In table 1, the countries considered in this review are LMICs recorded and identified by the World Bank. These are countries that have a gross national income per capita of less than US$4465.00.^15^ These countries include:

**Table 1:** Low- and middle-income countries^15^

Continent	Countries
Africa	Algeria, Angola, Benin, Botswana, Burkina Faso, Burundi, Cabo Verde, Cameroon, Central African Republic, Chad, Comoros, Congo, Dem Rep., Congo, Rep., Côte d'Ivoire, Djibouti, Egypt, Arab Rep., Eritrea, Eswatini, Ethiopia, Gambia, The, Ghana, Guinea, Guinea-Bissau, Kenya, Lesotho, Liberia, Madagascar, Malawi, Mali, Mauritania, Mauritius, Morocco, Mozambique, Namibia, Niger, Nigeria, Rwanda, São Tomé and Príncipe, Senegal, Sierra Leone, Somalia (Fed. Rep.), South Africa, South Sudan, Sudan, Tanzania, Togo, Tunisia, Uganda, Zambia, Zimbabwe
Asia	Afghanistan, Armenia, Bangladesh, Bhutan, Cambodia, China, India, Indonesia, Iran (Islamic Rep.), Iraq, Jordan, Korea (Dem. People's Rep.), Kyrgyz Republic, Lao PDR, Lebanon, Malaysia, Maldives, Mongolia, Myanmar, Nepal, Pakistan, Philippines, Sri Lanka, Syrian Arab Republic, Tajikistan, Thailand, Timor-Leste, Uzbekistan, Viet Nam, Yemen, Rep.
Europe	Albania, Belarus, Bosnia and Herzegovina, Georgia, Kosovo, Moldova, Montenegro, North Macedonia, Serbia, Ukraine, Türkiye
North America	Belize, Cuba, Dominican Republic, El Salvador, Grenada, Guatemala, Haiti, Honduras, Jamaica, Mexico, Nicaragua, St. Lucia, St. Vincent and the Grenadines
Oceania	Fiji, Kiribati, Marshall Islands, Micronesia (Fed. Sts.), Papua New Guinea, Samoa, Solomon Islands, Tonga, Tuvalu, Vanuatu
South America	Argentina, Bolivia, Brazil, Colombia, Ecuador, Guyana, Paraguay, Peru, Suriname

### Statement of the research goals

The goal of the literature search was to obtain research literature that involved studies conducted within the last 10 years in peer-reviewed publications regarding LMICs that used AI technologies or algorithms in the risk assessment of hypertension. The literature search strategy that was developed and implemented in this scoping review was based on the Population, Concept and Context (PCC) framework. Table 2 details how the PCC framework was applied to aid in the search for literature.

**Table 2:** Framework used for literature search strategy

PCC	Description
Population	Adult population suffering from hypertension within LMICs aged 18 years and older
Concept	Application of artificial intelligence in prediction or risk assessment
Context	LMICs context settings

LMICs, low- and middle-income countries.

Medical Subject Headings (MeSH) terms used to find literature in the search databases include ‘Hypertension’, ‘Blood Pressure’ and ‘Cardiovascular Diseases’. Other keywords used include ‘Systolic Pressure’ and ‘Diastolic Pressure’ to capture relevant studies. Research literature was spooled from the selected search databases using different combinations of the following key concepts (in bold font) and alternative terms found in table 3.

**Table 3:** Literature search terms

**Hypertension**	**Lower-middle-income countries**	**Artificial intelligence**
Hypertension Hypertension Disease Hypertension Risk Blood pressure Cardiovascular Disease Systolic Pressure Diastolic Pressure	Lower middle-income countries Low-income countries Algeria, Angola, Benin, Botswana, Burkina Faso, Burundi, Cabo Verde, Cameroon, Central African Republic, Chad, Comoros, Congo, Dem Rep., Congo, Rep., Côte d'Ivoire, Djibouti, Egypt, Arab Rep., Eritrea, Eswatini, Ethiopia, Gambia, The, Ghana, Guinea, Guinea-Bissau, Kenya, Lesotho, Liberia, Madagascar, Malawi, Mali, Mauritania, Mauritius, Morocco, Mozambique, Namibia, Niger, Nigeria, Rwanda, São Tomé and Principe, Senegal, Sierra Leone, Somalia (Fed. Rep.), South Africa, South Sudan, Sudan, Tanzania, Togo, Tunisia, Uganda, Zambia, Zimbabwe Afghanistan, Armenia, Bangladesh, Bhutan, Cambodia, China, India, Indonesia, Iran (Islamic Rep.), Iraq, Jordan, Korea (Dem. People’s Rep.), Kyrgyz Republic, Lao PDR, Lebanon, Malaysia, Maldives, Mongolia, Myanmar, Nepal, Pakistan, Philippines, Sri Lanka, Syrian Arab Republic, Tajikistan, Thailand, Timor-Leste, Uzbekistan, Viet Nam, Yemen, Rep., Albania, Belarus, Bosnia and Herzegovina, Georgia, Kosovo, Moldova, Montenegro, North Macedonia, Serbia, Ukraine, Türkiye, Belize, Cuba, Dominican Republic, El Salvador, Grenada, Guatemala, Haiti, Honduras, Jamaica, Mexico, Nicaragua, St. Lucia, St. Vincent and the Grenadines Fiji, Kiribati, Marshall Islands, Micronesia (Fed. Sts.), Papua New Guinea, Samoa, Solomon Islands, Tonga, Tuvalu, Vanuatu, Argentina, Bolivia, Brazil, Colombia, Ecuador, Guyana, Paraguay, Peru, Suriname	Machine Learning (ML) Deep Learning Neural Networks Optimization Algorithms Algorithm AI ML Supervised learning Unsupervised learning Semi-supervised learning

### Statement of the search strategy

Two independent reviewers used the search terms in the online supplemental file to identify relevant literature. Initial scans were followed by a detailed evaluation based on well-structured and clear inclusion and exclusion criteria. A third, more experienced reviewer resolved any selection discrepancies flagged from the first two reviews. Data from the final list were extracted and compiled into a Microsoft Excel file for analysis, including the following:

10.1136/bmjph-2025-003435.supp1 Supplementary data

1. Name of author
2. Year of publication
3. Country in which the study is done
4. Title of literature
5. Study objectives
6. Type of artificial intelligence algorithm(s) used
7. Characteristics of the study population
8. Sample size
9. Sampling method
10. Study design
11. Study context
12. Key findings/results
13. Strengths and weaknesses of the study

### Statement of the inclusion and exclusion criteria

The inclusion and exclusion criteria defined and used to guide the literature selection process within this review included the following:

1. The literature should primarily be about the study of the deployment of AI algorithms or tools in the diagnosis, detection or risk assessment of hypertension in an LMIC.
2. The data used in the literature to train and assess any implemented AI algorithms should be obtained from an LMIC.
3. The literature should have been published within the last 10 years, that is, 2015–2025.

## Results

**Figure 1:**
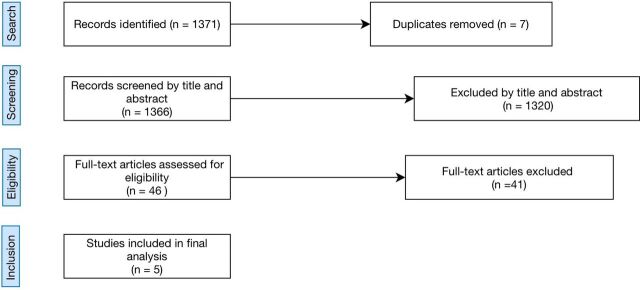
Flow chart for study search and screening.

Figure 1 shows the flow chart for study search and screening the studies. The literature search using the selected databases resulted in 1371 papers with 7 duplicates initially removed, resulting in 1366 papers. The next phase involved reviewing the titles and abstracts of these papers related to the subject review. After this stage, 1320 papers did not make the cut, leaving 46 papers. The last screening stage involved further review of the content in the papers using the inclusion criteria outlined in the Methods section. Therefore, a total of five studies were included in the final analysis.

### Statement of the characteristics of interest of the final papers

After conducting the review, five papers were selected based on the inclusion and exclusion criteria. All papers strictly considered the application of AI in hypertension risk assessment and prediction in LMICs. Algorithms commonly reported across these studies include logistic regression, decision trees and naïve Bayes. Each study used local datasets, and all were conducted from 2021 onwards. A summary of the selected studies can be found in table 4.

**Table 4:** Summary of studies selected

Authors	Country of study	Title of literature	Study objectives	Artificial intelligence algorithms	Characteristics of study population	Sample size	Study design	Strengths	Weaknesses
Md. Merajul Islam *et al*^16^	Ethiopia	Predicting the risk of hypertension using machine learning algorithms: a cross-sectional study in Ethiopia	Develop a machine learning system to predict patients at risk of hypertension in Ethiopia.	Boruta feature selection, logistic regression, ANN, random forest, XGBoost, SHAP	Adults aged 31–90 years (hypertensive patients); community-based sample (Hawassa city); both sexes	612	Cross-sectional study	XGBoost achieved high predictive accuracy. The Boruta feature selection method enhanced robustness.	Sample limited to permanent residents; area excluded those under 30 years old.
Sirinat Wanriko *et al*^19^	Kenya	Risk Assessment of Pregnancy-Induced Hypertension Using a Machine Learning Approach	Develop a predictive model for pregnancy-induced hypertension using ML algorithms.	Logistic regression, kNN, Decision Tree, Random Forest, MLP, SVM, Naive Bayes	Not reported (NR); female participants only (pregnant women); hospital-based sample (Nairobi)	352	Experimental study	Random forest achieved the highest accuracy (89.62%) on balanced data; AI assists early assessment.	Small sample size (25% of women had pre-eclampsia); limited generalisability.
Md. Asadullah *et al*^17^	Bangladesh	Evaluation of machine learning techniques for hypertension risk prediction based on medical data in Bangladesh	Improve NCD intervention strategies through ML-based risk prediction and data analysis.	Naive Bayes, SVM, logistic regression, random forest	NR; both genders; hospital-based sample–medical data from a private hospital (2014–2015)	9620	Retrospective study	Combines multiple ML algorithms to improve accuracy; the ROC curve is used to assess results.	Limited references to successful prior models; reliability is reduced when using single algorithms.
Sheikh Mohammed Shariful Islam *et al*^18^	Bangladesh, Nepal and India	Machine Learning Approaches for Predicting Hypertension and Its Associated Factors Using Population-Level Data from Three South Asian Countries	Compare ML models for predicting hypertension and associated factors using cross-national datasets.	Decision Tree, Random Forest, GBM, XGBoost, Logistic Regression, LDA	Males aged 15–54 and females aged 15–49; National-level dataset - Demographic and Health Survey in Bangladesh, Nepal and India	818 603	Retrospective study	ML models predicted hypertension with high accuracy using non-invasive data; they identified key risk factors (age, BMI).	Limited variables; some data from 2016; ML models have limits in causal inference.
Damilare T. Daramola *et al*^20^	Nigeria	Advancing Hypertension Risk Prediction in African Healthcare: A ML Approach with Web-Based Visualization and Interpretability	Develop a Web-based ML-powered platform for hypertension risk prediction	Logistic Regression, SVM, Decision Tree	NR; both genders; community-based sample (through outreaches)	800	Retrospective Study	97% accuracy with decision tree; real-time web app; Seamless integration with EHR	Small dataset; potential overfitting; varying acceptance of ML-based healthcare solutions due to cultural influences

AI, artificial intelligence; ANN, artificial neural network; BMI, body mass index; EHR, electronic health record; GBM, Gradient Boosting Machine; kNN, k-Nearest Neighbours; LDA, linear discriminant analysis; ML, machine learning; MLP, Multilayer Perceptron; NCD, non-communicable disease; ROC, receiver operating characteristic curve; SHAP, SHapley Additive exPlanations; SVM, support vector machine; XGBoost, extreme gradient boosting.

## Discussions

### AI-based hypertension prediction in LMICs: current evidence

The following studies prove that work is being done in predicting and diagnosing hypertension using AI. Islam *et al* looked at predicting the risk of hypertension using machine learning algorithms. In Islam *et al*, the authors analysed data from 612 Ethiopian HPT respondents using four models (logistic regression, artificial neural network, random forest and extreme gradient boosting (XGB)). The XGB model was selected as it achieved a prediction accuracy of 88.81% in predicting hypertension risk in patients.^16^ Similarly, Asadullah *et al* applied machine learning models such as naive Bayes, support vector machine, logistic regression and random forest by evaluating machine learning techniques for hypertension risk prediction based on medical data. In this study, the hybrid model, created by stacking naive Bayes, support vector machine, logistic regression and random forest, achieved a prediction of 78.17%, making it the top-performing model.^17^ Building on the work done by Asadullah *et al*, research by Islam *et al* involving HPT patients in South Asian countries (Bangladesh, Nepal and India) revealed that non-invasive information, using algorithms such as decision trees, random forest algorithm and boosting machines, can predict hypertension with high accuracy.^18^ Notably, Wanriko *et al*’s work done in Kenya highlights ongoing efforts to provide early diagnosis of pregnancy-induced hypertension to prevent pregnancy-induced hypertension, complications and death of pregnant women.^19^ Additionally, Damilare developed a risk prediction tool using data from community-based outreaches in Nigeria. Using the decision tree model, the AI solution was able to predict patient hypertension risk level with an outstanding accuracy of 97%.^20^ The risk prediction system is web-based, making it accessible to healthcare workers and patients for on-demand risk assessment and patient management. According to its developers, the platform is designed to be scalable and adaptable to varying healthcare ecosystems.^20^ These studies show that AI can potentially reduce the increasing rate of HPT patients in LMICs using early-stage prediction.

### Some challenges affecting AI implementation in LMICs

After conducting this review, it is apparent that health ecosystems in LMICs are now having conversations regarding AI in health. Unfortunately, some hindrances slow down the implementation of AI tools in LMICs. A few reasons have been highlighted below:

#### Lack of quality data

A key challenge limiting the effective use of AI for hypertension prediction in LMICs is the lack of high-quality, context-specific data. Digital medical systems and electronic health records are not widely adopted in many countries, resulting in datasets that are incomplete, inconsistent or unavailable for training AI models.^21^ When AI systems are trained on poor-quality data, they replicate the gaps and inconsistencies in those datasets.^22^ Yu and Zhai describe this as the *AI Deployment Paradox*, which leads to low-performing models that may amplify disparities rather than improve health outcomes.^22^

The situation worsens as there is heavy dependence on datasets from high-income countries (HICs), where most AI and hypertension research (approximately 75%–80%) is conducted.^23^ These studies use high-quality datasets from electronic medical record systems or population-level biobanks.^24^ On the other hand, LMIC studies, including those in this review, are fewer in number and constrained by inconsistent health data, small sample sizes and limited digital capacity (as shown in table 4).^24^ While HIC models often achieve high accuracy, their applicability to LMICs is impeded by differences in data quality, context and population characteristics.^25^ These insights justify the need for locally developed high-quality datasets and context-appropriate approaches.

#### Restrictions in infrastructure

LMICs face the issue of old infrastructure coupled with limited technological support. Current IT systems do not have the capacity or bandwidth to support AI applications and tools, including reliable internet connectivity, information management systems, adaptable software, data storage and strong data privacy regulation. Due to this poor infrastructure, AI applications cannot be scaled and implemented widely because of their limits in extendibility and adaptability.^26^

#### Limited AI expertise in LMICs

A major constraint to AI development in LMICs is the limited number of trained professionals capable of building, managing and using AI systems effectively. Many health systems do not have enough personnel with the technical skills required to support AI tools, which restricts current service delivery and slows future innovation. Unfortunately, skilled workers are moving to more economically prosperous countries, further affecting the local health ecosystem. This has resulted in a decrease in skilled personnel needed to innovate and create tailored and contextualised AI solutions for the unique desires of the local population. Brain drain severely affects marginalised populations with limited access to care, where AI could be utilised to optimise resources. The ongoing loss further weakens already strained healthcare ecosystems and limits the ability to create context-specific AI applications.^27^

#### Limited awareness of AI models

Many LMICs have limited awareness and understanding of AI models, possibly due to national AI policies and readiness strategies not being recognised. The absence of a clear framework obstructs AI adoption and contributes to low levels of trust in AI systems. There is also a lack of training and education on the benefits and areas of opportunity in AI, leading to limited awareness. When it comes to ethical, regulatory and funding concerns for ML technologies, governments and policymakers do not see it as a priority in LMICs, where short-term local needs take precedence. These challenges can drive a low awareness of AI and a negative perception of it, leading to resistance to AI adoption.^21^

#### Corruption as a barrier to AI deployment

Corruption further obstructs AI adoption in LMICs. Weak and non-transparent governance systems allow corrupt officials to benefit from practices such as bribery, diversion of funds and fraud, as noted by the *U4-Anti Corruption Resource Centre*. AI and machine-learning technologies have the potential to improve transparency, strengthen accountability and reduce opportunities for misconduct. Hence, their implementation may threaten the interests of individuals who benefit from existing governance downfalls. Therefore, corruption can directly discourage efforts to introduce AI systems that would rather increase performance in public services and reduce corruption risks.^28^

### Some solutions to mitigate challenges in the implementation of AI in LMICs

Though there are setbacks, there are effective solutions for LMICs to mitigate the challenges of technology interventions in predicting, preventing and monitoring hypertension.

#### Data collection solution

For data collections to be optimal, research organisational bodies in LMICs should have standardised metrics for data quality. Primary or front-line care workers should be involved in the data collection process to make it a clinical practice. These can be implemented by training stakeholders in the data collection process, especially in understanding data sources and their context. Investment should be put into relatively low-cost technologies and public health data from non-traditional sources such as SMS-based health surveys, community health worker reports and social media, among others. Promotion on creating and sharing open-source databases in addition to policies and investment in data collection and standardisation can lead to an efficient data collection process resulting in high-quality data.^21^

#### Storage and infrastructure solution

A key requirement for effective AI deployment in LMICs is adequate storage capacity and supportive digital infrastructure. Investing in the nationwide distribution of internet connectivity to the public is one of the first crucial steps to this deployment. López *et al* suggest LMICs should be intentional about seeking out programmes and funding by IT industries, providing low-cost infrastructure and computer capacity for data storage. López *et al* note that sustained funding in digital infrastructure requires prioritising research and development of open-sourced tools and resources, which can help attract interest from foreign investors and international organisations.^21^

Greater internet access increases the reach of digital health platforms even in rural communities. However, limited internet access and the high cost of broadband restrict digital health solutions in LMICs. The *Broadband Commission’s Promise of Digital Health* Report (2018) highlights several strategies that governments can implement to improve digital access, including constructing public access points, creating incentives for telecommunication providers to expand to underserved areas, and promoting infrastructure sharing to reduce costs.^29^

#### AI application design solution

To increase engagement and usability of these AI/ML solutions, human-centred design methodologies should be introduced from inception, including stakeholders such as governments, healthcare providers and other key actors. In addition, the inputs of women, minorities and poor communities should be prioritised to create multifaceted AI/ML solutions. These solutions should also include interoperability features to allow systems to communicate and benefit from collaboration between LMIC and HIC developers, particularly in building open-source digital health applications.^21^

The *Broadband Commission’s Promise of Digital Health* Report (2018) noted that digital health tools can only be helpful if tailored to the user’s specific needs. The end user’s input must be incorporated from initiation and continuously throughout the digital platform’s development. Design features that enhanced user engagement include the implementation of behavioural science teachings instead of one-size-fits-all content, human interactivity, user feedback influencing decision-making, and co-creation and designing with health target users.^29^ Strengthening digital health strategies also requires training workers in essential digital skills. These range from basic operational abilities like connecting a device to the internet to functional skills such as entering or sharing patient data and advanced skills (eg, programming a medical device software, running analytics for health data).^29^

Collectively, these approaches help address the core barriers limiting AI deployment in LMICs. By intentionally involving government officials, policymakers, healthcare providers and community members in the design and implementation process, these solutions strengthen engagement and build trust in AI technologies. Capacity building initiatives and digital skills training improve AI literacy and awareness. In addition, increased transparency and participation throughout the design and implementation may reduce mitigation risks by ensuring decision-makers are actively participating in and accountable for the deployment process. These coordinated efforts foster more collaborative, appropriate and contextually relevant AI systems that are better aligned with the needs of LMIC health ecosystems.

## Limitations and strengths

Out of all the papers found for this scoping review, only a few published papers strictly met our inclusion-exclusion criteria and applied AI in the prediction and risk assessment of hypertension in LMICs.

Though we found numerous papers regarding the search criteria, only five fit the exclusion-inclusion criteria. This is due to limited work being done using data authentically from LMICs. Research papers involving LMICs use data from HICs; they are not to blame, as data are not readily available. Hence, the only viable option is to use simulated data from HICs. However, as discussed earlier, this may be detrimental to the area where the AI tool tries to help because it does not provide a specifically tailored product for the local user.^25^ One way to overcome this challenge is by promoting the sharing and creation of local open-source databases.^21^ Local research organisations, the government and policymakers can take on this initiative for effective implementation.^21^

This study could have expanded its literature search to include studies in languages other than English. Despite this limitation, this review is one of the first studies to provide a review of the published literature on the applications of AI in hypertension prediction and risk assessment in LMICs. Future studies should use multilingual AI applications to aid in literature reviews and analysis, therefore reducing language bias in research.^23^

Despite these limitations, to the best of our knowledge, this work is one of the first few studies to provide a review on published literature of the applications of AI in hypertension prediction and risk assessment in LMICs.

## Conclusions

The studies have shown significant potential to be adopted in LMICs, which will, in turn, aid in preventing hypertension before it escalates. Implementing an AI tool for predicting hypertension could result in early onset detection and intervention, minimising treatment costs and mortality rates. With this proactive strategy, fewer individuals develop chronic hypertension, which, in turn, alleviates the economic burden of high treatment costs for LMICs.

The capability of AI to evaluate vast amounts of data and produce precise predictions is priceless, especially in resource-constrained settings where the prevalence of hypertension is increasing by the day. Though there is potential, there are challenges in implementing AI systems. These challenges include models customised to local contexts, limited data available and implementing AI tools into healthcare systems. Future research should concentrate on models that can be deployed in the context of LMICs’ healthcare systems.

The application of AI in intervening in hypertension in LMICs has great potential. However, this idea can only come to fruition with locally contextualised AI models and readily available and validated local data resources.

## Data Availability

All data relevant to the study are included in the article. The data analysed consists of previously published, publicly available research articles identified through database searches (PubMed, Elsevier, Google Scholar, Scopus, IEEE, and ACM Digital Library). No new primary data were generated during this study. The extracted datasets—comprising study characteristics, algorithm descriptions, and outcome summaries—were compiled for the purpose of this scoping review. Because the review relies entirely on secondary, published sources, there are no individual participant data or confidential materials involved.

## References

[1] Hypertension. Available: https://www.who.int/news-room/fact-sheets/detail/hypertension [Accessed 19 Mar 2024].

[2] Moloro AH, Seid AA, Jaleta FY. A systematic review and meta-analysis protocol on hypertension prevalence and associated factors among bank workers in Africa. SAGE Open Med 2023;11:20503121231172001. 10.1177/20503121231172001 37181276 PMC10170600

[3] Bosu WK, Reilly ST, Aheto JMK, et al. Hypertension in older adults in Africa: A systematic review and meta-analysis. PLoS One 2019;14:e0214934. 10.1371/journal.pone.0214934 30951534 PMC6450645

[4] Piette JD, Datwani H, Gaudioso S, et al. Hypertension Management Using Mobile Technology and Home Blood Pressure Monitoring: Results of a Randomized Trial in Two Low/Middle-Income Countries. Telemedicine and E-Health 2012;18:613–20. 10.1089/tmj.2011.0271 23061642 PMC4361160

[5] Stephen BU-A, Uzoewulu BC, Asuquo PM, et al. Diabetes and hypertension MobileHealth systems: a review of general challenges and advancements. J Eng Appl Sci 2023;70:1. 10.1186/s44147-023-00240-6

[6] Gafane-Matemane LF, Mokwatsi GG, Boateng D. Hypertension management in sub-saharan africa: an overview of challenges and opportunities for telemedicine.

[7] Jiang Q, Gong D, Li H, et al. Development and Validation of a Risk Score Screening Tool to Identify People at Risk for Hypertension in Shanghai, China. Risk Manag Healthc Policy 2022;15:553–62. 10.2147/RMHP.S354057 35386277 PMC8977866

[8] Ordunez P, Tajer C, Gaziano T, et al. The HEARTS app: a clinical tool for cardiovascular risk and hypertension management in primary health care. Rev Panam Salud Publica 2022;46:e12. 10.26633/RPSP.2022.12 35355690 PMC8959249

[9] What is artificial intelligence (AI)? | IBM. Available: https://www.ibm.com/topics/artificial-intelligence [Accessed 26 Mar 2024].

[10] Yazdi M, Zarei E, Adumene S, et al. Navigating the Power of Artificial Intelligence in Risk Management: A Comparative Analysis. Safety 2024;10:42. 10.3390/safety10020042

[11] ‘Artificial intelligence (AI): what it is and how it is used’, investopedia. Available: https://www.investopedia.com/terms/a/artificial-intelligence-ai.asp [Accessed 26 Mar 2024].

[12] Barker J, Sau A, Pastika L, et al. 206 Artificial intelligence–enabled electrocardiography for hypertension diagnosis. Heart 2024.:A217. 10.1136/heartjnl-2024-BCS.201

[13] Soh DCK, Ng EYK, Jahmunah V, et al. A computational intelligence tool for the detection of hypertension using empirical mode decomposition. Comput Biol Med 2020;118:103630. 10.1016/j.compbiomed.2020.103630 32174317

[14] Tricco AC, Lillie E, Zarin W, et al. PRISMA Extension for Scoping Reviews (PRISMA-ScR): Checklist and Explanation. Ann Intern Med 2018;169:467–73. 10.7326/M18-0850 30178033

[15] World Bank Open Data. World bank open data. Available: https://data.worldbank.org [Accessed 19 Mar 2024].

[16] Islam MM, Alam MJ, Maniruzzaman M, et al. Predicting the risk of hypertension using machine learning algorithms: A cross sectional study in Ethiopia. PLoS One 2023;18:e0289613. 10.1371/journal.pone.0289613 37616271 PMC10449142

[17] Asadullah Md, Hossain MdM, Rahaman S, et al. Evaluation of machine learning techniques for hypertension risk prediction based on medical data in Bangladesh. IJEECS 2023;31:1794. 10.11591/ijeecs.v31.i3.pp1794-1802

[18] Islam SMS, Talukder A, Awal MA, et al. Machine Learning Approaches for Predicting Hypertension and Its Associated Factors Using Population-Level Data From Three South Asian Countries. Front Cardiovasc Med 2022;9:839379. 10.3389/fcvm.2022.839379 35433854 PMC9008259

[19] Wanriko S, Hnoohom N, Wongpatikaseree K, et al. Risk assessment of pregnancy-induced hypertension using a machine learning approach. 2021 Joint International Conference on Digital Arts, Media and Technology with ECTI Northern Section Conference on Electrical, Electronics, Computer and Telecommunication Engineering; Cha-am, Thailand, March 2021:233–7. 10.1109/ECTIDAMTNCON51128.2021.9425764

[20] Damilare D. Advancing Hypertension Risk Prediction in African Healthcare: A Machine Learning Approach with Web-Based Visualization and Interpretability. J AI & Mach Lear 2025;1:1–8.

[21] López DM, Rico-Olarte C, Blobel B, et al. Challenges and solutions for transforming health ecosystems in low- and middle-income countries through artificial intelligence. Front Med (Lausanne) 2022;9:958097. 10.3389/fmed.2022.958097 36530888 PMC9755337

[22] Yu L, Zhai X. Use of artificial intelligence to address health disparities in low- and middle-income countries: a thematic analysis of ethical issues. Public Health (Fairfax) 2024;234:77–83. 10.1016/j.puhe.2024.05.029 38964129

[23] B L. Artificial Intelligence in Hypertension: Trends, Outcomes, and Collaborations (2013– 2023). Med Clin Res 2025;10:01–8. 10.33140/MCR.10.03.05

[24] Meder B, Asselbergs FW, Ashley E. Artificial intelligence to improve cardiovascular population health. Eur Heart J 2025;46:1907–16. 10.1093/eurheartj/ehaf125 40106837 PMC12093147

[25] Ciecierski-Holmes T, Singh R, Axt M, et al. Artificial intelligence for strengthening healthcare systems in low- and middle-income countries: a systematic scoping review. NPJ Digit Med 2022;5:162. 10.1038/s41746-022-00700-y 36307479 PMC9614192

[26] Labrique AB, Wadhwani C, Williams KA, et al. Best practices in scaling digital health in low and middle income countries. Global Health 2018;14:103. 10.1186/s12992-018-0424-z 30390686 PMC6215624

[27] Pillai AS. Artificial Intelligence in Healthcare Systems of Low- and Middle-Income Countries: Requirements, Gaps, Challenges, and Potential Strategies. International Journal of Applied Health Care Analytics 2023;8:19–33. Available: https://norislab.com/index.php/IJAHA/article/view/72

[28] Corruption and Anti-Corruption within Social Protection Systems in Low- and Middle-Income Countries (LMICs). U4 anti-corruption resource centre. Available: https://www.u4.no/publications/corruption-and-anti-corruption-within-social-protection-systems-in-low-and-middle-income-countries-lmics [Accessed 26 Nov 2024].

[29] The promise of digital health: addressing non-communicable diseases to accelerate universal health coverage in lmics. Novartis Foundation. Accessed: Jan; 2025. Available: https://www.novartisfoundation.org/news/media-library/promise-digital-health-addressing-non-communicable-diseases-accelerate-universal-health-coverage-lmics

