# Supplementary material for: Can artificial intelligence bridge critical gaps in hypertension prediction and risk assessment in low- and middle-income countries? A scoping review
Source: BMJ Public Health. 2026 May 22;4(2):e003435. doi: 10.1136/bmjph-2025-003435 (PMC13202181; doi:10.1136/bmjph-2025-003435)
Supplement: Supplementary data [file bmjph-4-2-s001.pdf]

APPENDIX A

Additional terms for the search strategy that can be used across the various search databases would include:

1. (("Hypertension Treatment" OR "High Blood Pressure" OR "Primary Hypertension" OR "Secondary Hypertension" OR "Hypertension Causes" OR "Hypertension Symptoms" OR "Hypertension Complications" OR "Hypertension Medications" OR "Hypertension Prevention" OR "Hypertension Diagnosis" OR "Hypertension Lifestyle Changes" OR "Hypertension Diet" OR "Hypertension Exercise" OR "Hypertension Monitoring" OR "Hypertension Awareness" OR "Hypertension Research" OR "Hypertension Control" OR "Hypertension Education") AND ("machine learning" OR "deep learning" OR "artificial intelligence" OR "neural networks") AND ("West Africa" OR "Africa" OR "Sub-saharan Africa")) AND (("Reinforcement Learning" OR "Convolutional Neural Networks (CNN)" OR "Recurrent Neural Networks (RNN)" OR "Generative Adversarial Networks (GANs)" OR "Support Vector Machines (SVM)" OR "Decision Trees" OR "Random Forests" OR "Gradient Boosting Machines (GBM)" OR "Natural Language Processing (NLP)" OR "Clustering Algorithms" OR "Dimensionality Reduction Techniques" OR "Ensemble Learning" OR "Transfer Learning" OR "Autoencoders" OR "Bayesian Methods" OR "K-means Clustering" OR "Markov Models" OR "Time Series Analysis" OR "Pattern Recognition" OR "Hyperparameter Tuning"))

2. (("Hypertension" OR "Hypertension Disease" OR "Hypertension Risk" OR "Blood Pressure" OR "Systolic Pressure" OR "Diastolic Pressure" OR "Cardiovascular Disease" OR "Heart Disease" OR "Hypertension in Low-Income Countries" OR "Hypertension in Lower-Middle-Income Countries" OR "Hypertension in Developing Countries" OR "Blood Pressure Variability" OR "Hypertension Risk Factors" OR "Blood Pressure Control" OR "Hypertension Epidemiology") AND ("Machine Learning" OR "Deep Learning" OR "Neural Networks" OR "Artificial Intelligence" OR "AI" OR "ML" OR "Predictive Modeling" OR "Risk Prediction Algorithms" OR "Medical Diagnostics" OR "Health Informatics" OR "Data Mining" OR "Supervised Learning" OR "Unsupervised Learning" OR "Semi-supervised Learning" OR "Reinforcement Learning" OR "Optimization Algorithms" OR "Algorithm Design" OR "Pattern Recognition" OR "Data-driven Healthcare" OR "Computational Biology") AND ("Low-Income Countries" OR "Lower-Middle-Income Countries" OR "Developing Nations" OR "Emerging Economies" OR "Resource-Limited Settings" OR "Global Health" OR "Public

Health in Africa" OR "Hypertension Management in Africa" OR "Africa" OR "Sub-Saharan Africa"))

3. (("Hypertension" OR "High Blood Pressure" OR "Cardiovascular Disease" OR "Hypertension in LMICs" OR "Blood Pressure Measurement" OR "Hypertension Risk Prediction" OR "Blood Pressure Trends" OR "Cardiovascular Risk Factors" OR "Hypertension Screening" OR "Hypertension Diagnosis" OR "Hypertension Complications" OR "Hypertension Prevention" OR "Cardiovascular Health" OR "Blood Pressure Management" OR "Blood Pressure Control Strategies") AND ("Machine Learning" OR "Deep Learning" OR "Neural Networks" OR "Artificial Intelligence" OR "AI" OR "ML" OR "Supervised Learning" OR "Unsupervised Learning" OR "Semi-supervised Learning" OR "Reinforcement Learning" OR "Neural Network Architectures" OR "Algorithm Development" OR "Health Data Analytics" OR "Clinical Decision Support Systems" OR "Predictive Analytics" OR "Personalized Medicine" OR "Medical Imaging Analysis" OR "Computational Techniques in Healthcare") AND ("Africa" OR "West Africa" OR "Sub-Saharan Africa" OR "Low-Income Countries" OR "Lower-Middle-Income Countries" OR "Developing Regions" OR "Global South" OR "Healthcare in Developing Countries" OR "Healthcare in LMICs" OR "Health Disparities"))

4. (("Hypertension" OR "Hypertension Disease" OR "Blood Pressure" OR "Cardiovascular Disease" OR "Systolic Blood Pressure" OR "Diastolic Blood Pressure" OR "Cardiovascular Health" OR "Hypertension Risk Factors" OR "Hypertension Detection" OR "Blood Pressure Monitoring" OR "Hypertension Control Strategies" OR "Blood Pressure Management" OR "Hypertension and Lifestyle" OR "Hypertension and Diet" OR "Hypertension and Exercise" OR "Cardiovascular Risk Assessment") AND ("Machine Learning" OR "Deep Learning" OR "Neural Networks" OR "Artificial Intelligence" OR "AI" OR "ML" OR "Predictive Modeling in Healthcare" OR "Medical Image Processing" OR "Algorithm Optimization" OR "Data Science" OR "Health Informatics" OR "Supervised Learning" OR "Unsupervised Learning" OR "Semi-supervised Learning" OR "Neural Network Models" OR "Feature Selection" OR "Big Data in Healthcare" OR "Clinical Algorithms") AND ("Low-Income Countries" OR "Lower-Middle-Income Countries" OR "Developing Countries" OR "Emerging Markets" OR "Sub-Saharan Africa" OR "West Africa" OR "Africa" OR "Global Health Challenges" OR "Public Health in Africa" OR "Chronic Disease Management in LMICs"))
